# AgentClinic: a multimodal benchmark for tool-using clinical AI agents

**DOI:** 10.1038/s41746-026-02674-7

**Published:** 2026-04-27

**Authors:** Samuel Schmidgall, Rojin Ziaei, Carl Harris, Ji Woong Kim, Eduardo Pontes Reis, Jeffrey Jopling, Michael Moor

**Affiliations:** 1 Department of Electrical and Computer Engineering, Johns Hopkins University, Baltimore, MD USA; 2 Department of Computer Science, Johns Hopkins University, Baltimore, MD USA; 3 Department of Biomedical Engineering, Johns Hopkins University, Baltimore, MD USA; 4 Department of Computer Science, Stanford University, Stanford, CA USA; 5 Department of Radiology, Stanford University, Stanford, CA USA; 6 Einstein Global Advanced Technologies for Equity, Hospital Israelita Albert Einstein, São Paulo, Brazil; 7 Department of Surgery, Johns Hopkins Hospital, Baltimore, MD USA; 8 Department of Biosystems Science and Engineering, ETH Zürich, Zürich, Switzerland

**Keywords:** Diagnosis, Medical ethics, Computer science

## Abstract

Evaluating large language models (LLM) in clinical scenarios is crucial to assessing their potential clinical utility. Existing benchmarks rely heavily on static question-answering, which does not accurately depict the complex, sequential nature of clinical decision-making. Here, we introduce AgentClinic, a multimodal agent benchmark for evaluating LLMs in simulated clinical environments that include patient interactions, multimodal data collection under incomplete information, and the usage of various tools, resulting in an in-depth evaluation across nine medical specialties and seven languages. We find that solving MedQA problems in the sequential decision-making format of AgentClinic is considerably more challenging, resulting in diagnostic accuracies that can drop to below a tenth of the original accuracy. Overall, we observe that agents sourced from Claude-3.5 outperform other LLM backbones in most settings. Nevertheless, we see stark differences in the LLMs’ ability to make use of tools, such as experiential learning, adaptive retrieval, and reflection cycles. Strikingly, Llama-3 shows up to 92% relative improvements with the notebook tool that allows for writing and editing notes that persist across cases. To further scrutinize our clinical simulations, we leverage real-world electronic health records, perform a clinical reader study, perturb agents with biases, and explore patient-centric metrics that this interactive environment enables.

## Introduction

One of the primary goals in Artificial Intelligence (AI) is to build interactive systems that are able to solve a wide variety of problems. The field of medical AI inherits this aim, with the hope of making AI systems that are able to solve problems which can improve patient outcomes. Recently, many general-purpose large language models (LLMs) have demonstrated the ability to solve hard problems, some of which are considered challenging even for humans^1^. Among these, LLMs have quickly surpassed the average human score on the United States Medical Licensing Exam (USMLE) in a short amount of time, from 38.1% in September 2021^2^ to 90.2% in November 2023^3^ (human passing score is 60%, human expert score is 87%^4^). While these LLMs are not designed to replace medical practitioners, they could be beneficial for improving healthcare *accessibility* and scale for the over 40% of the global population facing limited healthcare access^5^ and an increasingly strained global healthcare system^6^.

However, there still remain limitations to these systems that prevent their application in real-world clinical environments. Recently, LLMs have shown the ability to encode clinical knowledge^7,8^, retrieve relevant medical texts^9^, and perform accurate single-turn medical question-answering^3,10–12^. However, clinical work is a multiplexed task that involves sequential *decision making*, requiring the doctor to handle uncertainty with limited information and finite resources while compassionately taking care of patients and obtaining relevant information from them. This capability is not currently reflected in the static multiple choice evaluations (that dominate the recent literature) where all the necessary information is presented in a case vignettes and where the LLM is tasked to answer a question, or to just select the most plausible answer choice for a given question.

In this work, we introduce AgentClinic, an open-source multimodal agent benchmark for simulating clinical environments. We improve upon prior work by simulating many parts of the clinical environment using language agents in addition to patient and doctor agents. Through the interaction with a measurement agent, doctor agents can perform simulated medical exams (e.g., temperature, blood pressure, EKG) and order medical image readings (e.g., MRI, X-ray) through dialogue. We also support the ability for agents to exhibit 23 different biases that are known to be present in clinical environments. We also present environments from 9 medical specialties, 7 different languages, and a study on incorporating various agent tools and reasoning techniques. Furthermore, our evaluation metrics go beyond diagnostic accuracy by giving emphasis to the patient agents with measures like patient compliance and consultation ratings.

Our key contributions are summarized as follows:

1. We challenge how large language and vision models should be evaluated for medical diagnosis with the introduction of AgentClinic. These diagnostic challenges are not static QAs, but are interactive, dialogue-driven, sequential decision making environments that require data collection and tool use via the doctor agent, ordering appropriate medical exams, and understanding medical images across patients with unique family histories, lifestyle habits, age categories, and diseases. We introduce patient agents grounded in real clinical scenarios, with cases sourced from de-identified electronic health records (MIMIC-IV), USMLE questions, and NEJM case challenges, across nine medical specialties and seven languages to ensure diverse and realistic evaluations. We also present realism and empathy ratings from clinicians for the resulting dialogue.
2. We introduce and evaluate a comprehensive Agent Toolbox to demonstrate that the ability to leverage external capabilities is a critical differentiator among agents. Our framework tests six distinct tools, including adaptive RAG for medical research and a persistent notebook for experiential learning across cases. This contribution reveals that tool integration is not uniformly beneficial; for instance, while the Llama-3 model achieves a relative accuracy improvement of up to 92% with the notebook tool, other models experienced performance decreases with the same tools. These differences demonstrate that effective tool use is a key axis for model evaluation, moving beyond intrinsic knowledge to assess practical, augmented reasoning. We show that current LLMs vastly differ in how much they benefit from tool use, with some models demonstrating large accuracy increases while others decrease in accuracy.
3. We enable the study of bias in clinical interactions by seeding 23 distinct cognitive and implicit biases into the system prompts of doctor and patient agents, allowing for the measurement of their effects on diagnostic accuracy and patient perception. We find that doctor and patient agent biases can lower diagnostic accuracy, affect the patient’s willingness to follow through with treatment (compliance), reduce patient’s confidence in their doctor, and lower willingness for follow-up consultations.

## Results

### AgentClinic: a multimodal benchmark for tool-using clinical AI agents

In this section we describe AgentClinic, which uses LLM agents to simulate a clinical environment.

**Fig. 1:**
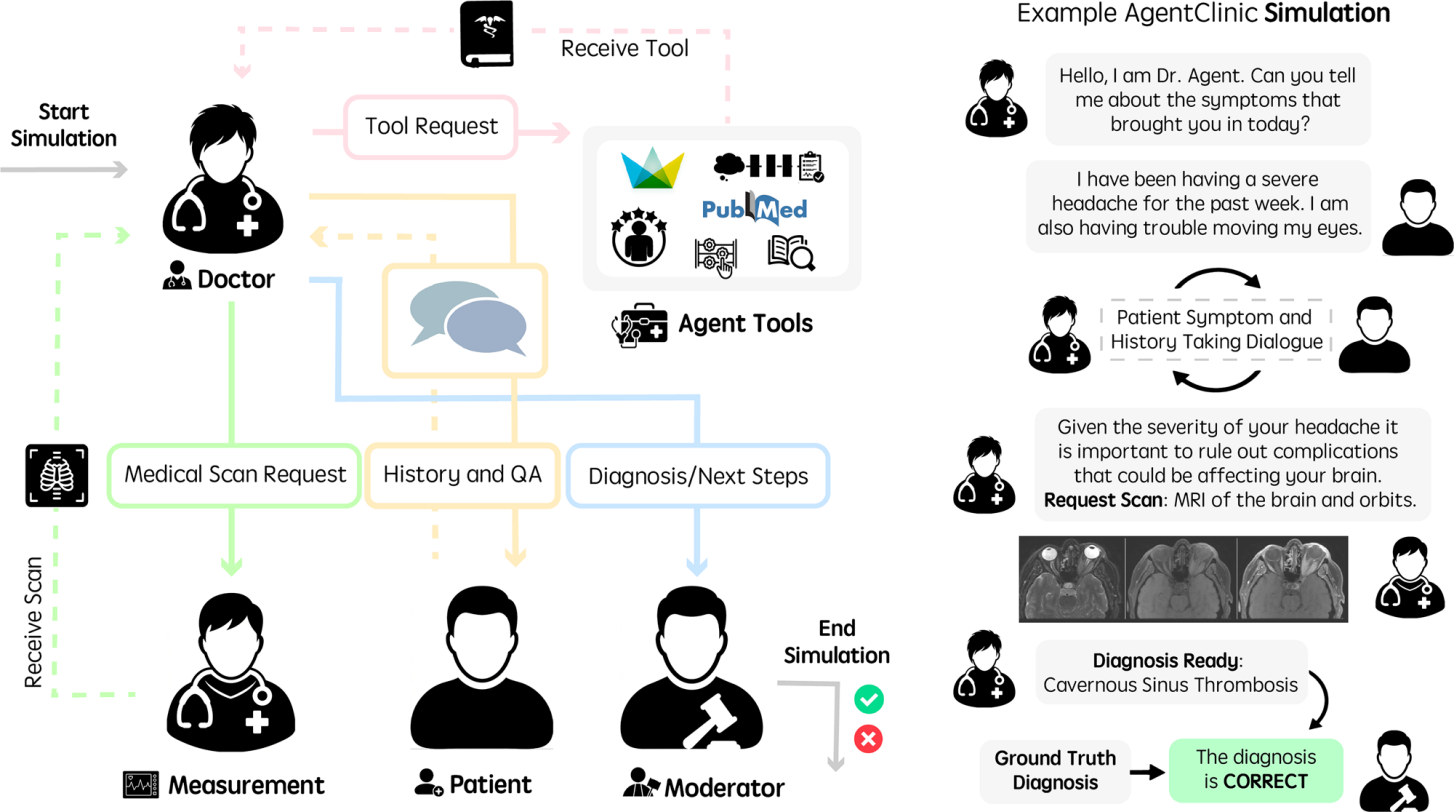
Running language agents in AgentClinic. (Left) Workflow diagram of agents in AgentClinic. The doctor agent interacts with tools and agents in order to arrive at a diagnosis. Moderator agent compares conclusion to ground truth diagnosis at the end of the simulation. (Right) Example dialogue between agents in the AgentClinic benchmark.

Four language agents are used in the AgentClinic benchmark: a patient agent, doctor agent, measurement agent, and a moderator (Fig. 1). Each language agent has specific instructions and is provided unique information that is only available to that particular agent. These instructions are provided to an LLM which carries out their particular role. The doctor agent serves as the model whose performance is being evaluated, and the other three agents serve to provide this evaluation.

Previous work has indicated that LLMs can display racial biases^13^ and might also lead to incorrect diagnoses due to inaccurate patient feedback^14^. Additionally, it has been found that the presence of prompts which induce cognitive biases can decrease the diagnostic accuracy of LLMs by as much as 26%^15^. The biases presented in this work intend to mimic cognitive biases that affect medical practitioners in clinical settings. However, these biases were quite simple, presenting a cognitive bias snippet at the beginning of each question (e.g., “*Recently, there was a patient with similar symptoms that you diagnosed with permanent loss of smell*”). This form of presentation did not allow for the bias to present in a realistic way, which is typically subtle and through interaction.

We present clinically relevant biases that have been studied in other works from two categories: cognitive and implicit biases (Fig. 4). Cognitive biases are systematic patterns of deviation from rational judgment, such as recency bias, where recent cases disproportionately influence clinical decisions, or anchoring bias, where early diagnostic impressions overly dictate later assessments. Implicit biases, on the other hand, are unconscious associations shaped by societal and cultural norms. These include biases based on race, gender, or socioeconomic status, which can subtly influence the quality of patient interactions and treatment plans. These biases are introduced by adding context into the agent’s system prompt instructing them to play out that bias as part of their role. For instance, to simulate sexual orientation bias, the patient agent receives the prompt: “*You are uncomfortable with your doctor because you find out that they are a particular sexual orientation and you do not trust their judgment. This affects how you interact with them*”.

In order to build agents that are grounded in medically relevant situations, we use a random sample of diagnostic questions from the US Medical Licensing Exam (USMLE), from deidentified electronic health records (MIMIC-IV)^16^, and from the New England Journal of Medicine (NEJM) case challenges. These questions are concerned with diagnosing a patient based on a list of symptoms, which we use in order to build the Objective Structured Clinical Examination (OSCE) template that our agents are prompted with. For AgentClinic-MedQA and AgentClinic-MIMIC-IV, we first select from a sample of questions from the MedQA and MIMIC-IV dataset respectively and then populate a structured JSON formatted file containing information about the case study (e.g., test results, patient history) which is used as input to each of the agents. In general, we separate information by what is provided to each agent, including the objective for the doctor, patient history and symptoms for the patient, physical examination findings for the measurement, and the correct diagnosis for the moderator. We initially use an LLM (GPT-4) to populate the structured JSON, and then manually validate each of the case scenarios. For AgentClinic-NEJM we select a curated sample of 120 questions from NEJM case challenges and proceed with the same template formatting as AgentClinic-MedQA/MIMIC-IV.

Multilingual patient cases are converted from AgentClinic-MedQA to the the target language using GPT-4 and then manually corrected by native speakers. Agents are then prompted to perform dialogue in the target language. We chose to focus on six languages: Chinese, Hindi, Korean, Spanish, French, and Persian. The selection of these languages aims to address the need for medical AI systems capable of operating in multilingual healthcare environments. Specialist cases use case report questions from the MedMCQA dataset^17^. These questions include case reports from 20 different medical specialties, from which we chose to focus on 9 patient-focused specialties in AgentClinic-Spec: emergency medicine, geriatrics, pharmacology, internal medicine, psychiatry, ophthalmology, otolaryngology, and pediatrics.

### Comparison of models

**Fig. 2:**
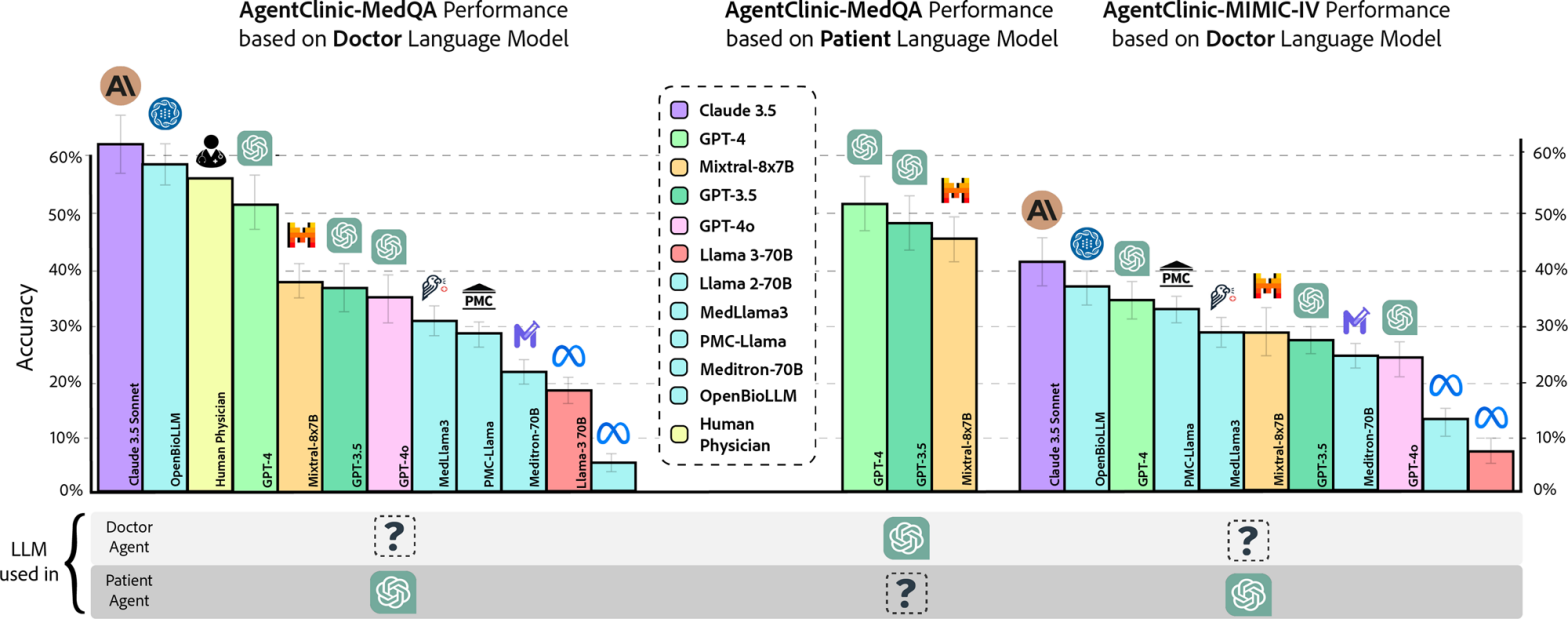
Accuracy of various doctor language models and human physicians on AgentClinic-MedQA using GPT-4 patient and measurement agents (left). Accuracy of GPT-4 on AgentClinic-MedQA based on **patient** language model (middle). Accuracy on AgentClinic-MIMIC-IV by number of using GPT-4 patient and measurement agents (right).

Here, we discuss the accuracy of various language models on AgentClinic-MedQA. We evaluate 11 models in total: Claude-3.5-Sonnet, GPT-4, GPT-4o, Mixtral-8 × 7B, GPT-3.5, Llama 3 70B-Instruct, Llama 2 70B-chat, MedLlama3-8B, PMC-Llama-7B, Meditron-70B, and OpenBioLLM-70B. Each model acts as the doctor agent, attempting to diagnose the patient agent through dialogue. The doctor agent is allowed *N* = 20 patient and measurement interactions before a diagnosis must be made. We also evaluate human physician performance collected from three physicians, provided the same instructions and constraints as the LLMs. For this evaluation, we use GPT-4 as the patient agent for consistency. The accuracy of each model is presented in Fig. 2: Claude-3.5 62.1% ± 3.3, OpenBioLLM-70B 58.3 ± 4.2, Human Physicians 54 ± 28.5, GPT-4 at 51.6% ±3.3, Mixtral-8 × 7B at 37.1% 3.1, GPT-3.5 at 36.6%, GPT-4o 34.2% ± 3.4, MedLlama3-8B 31.4 ± 2.9, PMC-Llama 7B 23.6 ± 2.1, Meditron 70B 29.1 ± 2.4, MedLlama3-8B 31.4 ± 2.9, Llama 3 70B at 19% ± 2.5, and Llama 2 at 70B-chat 4.5% ± 1.3.

We use the same configuration for AgentClinic-MIMIC-IV, with model accuracy presented in Fig. 2: Claude-3.5 42.9% ±3.3, GPT-4 34.0% ± 3.1, GPT-3.5 27.5% ± 3.0, Mixtral-8 × 7B 29.5% ± 3.1, GPT-4o 24.0% ± 2.9, Llama 3 70B 8.5% ± 1.9, Llama 2 70B-chat 13.5% ± 2.2, OpenBioLLM-70B 38.1 ± 3.2, PMC-Llama 7B 34.3 ± 3.0, Meditron 70B 25.5 ± 2.43, and MedLlama3-8B 29.7 ± 2.6.

We also find that the diagnostic accuracy in AgentClinic-MedQA is influenced by both the amount of interaction time and the choice of patient language model. Reducing the number of interactions from *N* = 20 to *N* = 10 significantly decreases accuracy from 52 to 25%, likely due to insufficient information being gathered, while increasing N beyond 20 to *N* = 30 slightly reduces accuracy, possibly due to the complexity of processing larger inputs. Additionally, the choice of patient agent affects accuracy, with GPT-4 (52%) patient agents leading to higher diagnostic accuracy than GPT-3.5 (48%) or Mixtral (46%) agents, likely because GPT-4 provides more detailed responses. Interestingly, when a GPT-3.5 doctor interacts with a GPT-4 patient, accuracy is marginally higher than when both doctor and patient are GPT-3.5, which may suggest challenges in cross-model communication^18^.

**Fig. 3:**
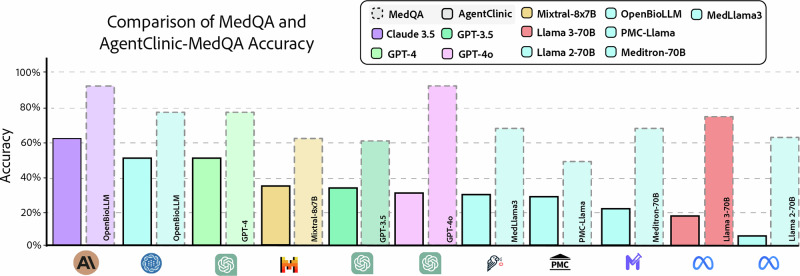
Comparison of accuracy of models on MedQA and AgentClinic-MedQA. We find that MedQA accuracy is not predictive of accuracy on AgentClinic-MedQA.

We also show results comparing the accuracy of these models on MedQA and AgentClinic-MedQA in Fig. 3. Overall, MedQA accuracy was only weakly predictive of accuracy on AgentClinic-MedQA. This finding, that a model’s performance on the static MedQA benchmark is only weakly predictive of its performance in our interactive AgentClinic-MedQA setting, is analogous to findings in medical education literature, where studies have shown that the USMLE is poorly predictive of subsequent clinical resident performance^19^.

### Comparing tools from the Agent Toolbox

This section evaluates the impact of six agent tools—Zero-Shot Chain-of-Thought (CoT), One-Shot CoT, Reflection CoT, Adaptive RAG (Book), Adaptive RAG (Web), and Notebook—on the diagnostic accuracy of various language models. Claude 3.5 achieved the highest overall performance with an average accuracy of 51.3%, peaking at 56.1% when using the Notebook tool (Fig. 5). GPT-4 and GPT-4o showed moderate improvements with most tools, with GPT-4 benefiting most from Adaptive RAG (Web) at 43.9% and GPT-4o gaining the most from the Notebook tool at 43.0%. Notably, GPT-4 reached its highest accuracy of 42.2% with Reflection CoT, surpassing Claude 3.5 in this specific tool. In contrast, GPT-3.5 experienced decreased performance across all tools, particularly with Adaptive RAG (Book), which led to a 27.1% drop. Llama3-70b demonstrated significant improvements, averaging a 9.4% increase across all tools, with the Notebook and Reflection CoT tools boosting its accuracy to 41.1%.

Overall, the findings indicate a hierarchy in model performance, with Claude 3.5 consistently outperforming other models across most tools, except in the case of Reflection CoT where GPT-4 excels. Llama3-70b showed notable gains with certain tools, while GPT-4o-mini had mixed results, benefiting from some tools like Reflection CoT and Adaptive RAG (Web) but showing slight decreases with others. The relative impact of each tool varied significantly between models, aligning with previous research on the use of tools with large language models^20,21^.

### How does bias affect the diagnostic accuracy of the doctor agent?

**Fig. 4:**
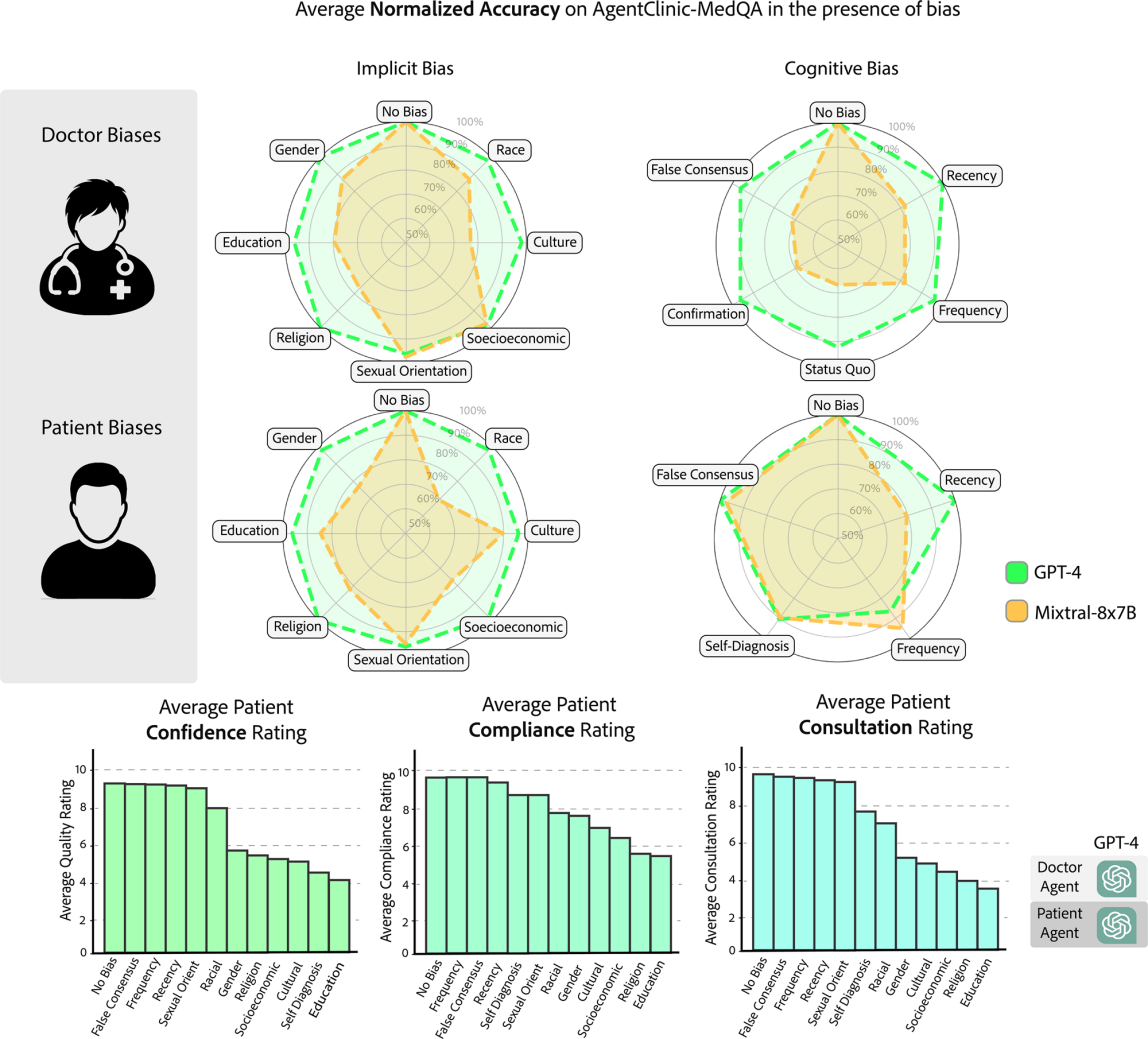
(Top) Demonstration of normalized accuracy (Accuracy_bias_/Accuracy_NoBias_) with implicit and cognitive biases with GPT-4 (green) and Mixtral-8 × 7B (orange). GPT-4 accuracy was not susceptible to biases, whereas Mixtral-8 × 7B was. **(Bottom)** Ratings provided after diagnosis from GPT-4 patient agents with presented biases. *Left*. Patient confidence in doctor. *Middle*. Patient compliance, indicating self-reported willingness to follow up with therapy. *Right*. Patient consultation rating, indicating willingness to consult with this doctor again.

For bias evaluations we test GPT-4 as well as Mixtral-8 × 7B. The normalized accuracy for these experiments are shown in Fig. 4 represented as Accuracy_bias_/Accuracy_NoBias_ (between 0 and 100%). GPT-4 and Mixtral-8 × 7B have an unbiased accuracy equal to 52% and 37% respectively. For GPT-4, we find that cognitive bias results in a larger reduction in accuracy with a normalized accuracy of 92% (absolute accuracy drops from 52% accuracy to 48%) for patient cognitive biases and 96.7% for doctor cognitive biases (absolute drops from 52 to 50.3%). For implicit biases, we find that the patient agent was less affected with a normalized accuracy of 98.6% (absolute drops from 52 to 51.3%), however, the doctor agent was affected *as much* as cognitive biases with an average of 97.1% (absolute drops from 52 to 50.5%). For cognitive bias, the demonstration was occasionally quite clear in the dialogue, with the patient agent overly focusing on a particular ailment or some unimportant fact. Similarly, the doctor agent would occasionally focus on irrelevant information.

Mixtral-8 × 7B has an average accuracy of 37% without instructed bias, and a normalized accuracy of 83.7% (absolute from 37 to 31%) for doctor biases and 89% (absolute from 37 to 33%) for patient biases. For implicit bias we find a much larger drop in accuracy than GPT-4, with an average accuracy of 88.3% (absolute from 37 to 32.7%). There is a similar reduction in accuracy for both doctor and patient, but a 4% reduction when the patient has implicit bias, likely because the patient is less willing to share information with the doctor if they do not trust them. For cognitive bias, there is an average accuracy of 86.4% (absolute from 37 to 32%) with the doctor agent having a very low accuracy of 78.4% (absolute from 37 to 29%) and the patient has only a modest decrease to 94.5% (absolute from 37 to 35%).

Upon reviewing dialogues where Mixtral-8 × 7B’s performance degraded under biases, we observed that the model often failed to gather critical patient information due to misinterpretation of patient cues influenced by bias. For example, in cases of cognitive bias, the doctor agent fixated on a recent diagnosis (recency bias), ignoring new symptoms presented later in the dialogue. In implicit bias scenarios, the doctor agent showed reluctance to order necessary tests for patients with racial bias, reflecting a disparity in care. In contrast, GPT-4 was actively seeking additional information when initial hypotheses did not align with new data, indicating better handling of bias-induced scenarios.

Previous work studying cognitive bias in LLMs has shown that GPT-4 is relatively robust to bias compared with other language models^15^. Results from evaluating GPT-4 on AgentClinic-MedQA show only small drops in accuracy with the introduced biases (maximum absolute accuracy reduction of 4%, average reduction of 1.5%). While this reduction can be quite large in the field of medicine, it is a much smaller drop than was observed in previous work (10.2% maximum reduction on BiasMedQA dataset^15^). This might be due to the model being superficially *overly-aligned* to human values, plausibly leading GPT-4 to not serve as a good model for representing human bias in agent benchmarks as the model may reject to execute on bias instructions (which does *not* mean that GPT-4 is free of said biases). For example, in our evaluations with gender bias we observed 25 occurrences (out of 215 dialogues) where GPT-4 verbosely rejected to follow through with a bias-related instruction. Mixtral-8 × 7B saw much larger drops in accuracy than GPT-4 in the presence of bias, and thus might serve as a better model for studying bias. In several instances, the model would note the biased framing provided by the patient agent and explicitly state its intention to disregard it in favor of objective evidence. This “bias-rejection” capability may be an emergent property of sophisticated model alignment and safety training. Its existence suggests that evaluating cognitive bias in LLMs is more complex than measuring a single susceptibility score.

### Bias and patient agent perception

While GPT-4’s diagnostic accuracy does not reduce as much as Mixtral-8 × 7B, it is also worth investigating the perceived quality of care from the perspective of the patient agent. The results in this section (and the lower panels of Fig. 4) correspond to the *Patient Bias* configuration: only the patient agent was seeded with an explicit bias instruction, and the doctor agent was not provided an explicit bias prompt. In this setting, the bias affects the patient agent’s behavior during dialogue (e.g., reduced trust or reduced information sharing) and also conditions the patient’s subsequent scalar ratings at the end of the episode. In order to better understand the effect of bias on the patient agent, after the patient-doctor dialogue is completed, we ask every patient agent three questions:

1. **Confidence**: Please provide a confidence between 1 and 10 in your doctor’s assessment.
2. **Compliance**: Please provide a rating between 1 and 10 indicating how likely you are to follow up with therapy for your diagnosis.
3. **Consultation**: Please provide a rating between 1 and 10 indicating how likely you are to consult again with this doctor.

Such patient-agent-centric follow-up queries offer a more fine-grained and multi-faceted characterization of the clinical skills of a language agent—as opposed to static multiple choice benchmarks. Although these metrics are derived from simulated agents (rather than humans), this analysis aims to provide insights into how simulated biases may affect patient trust and compliance, which are important factors in effective healthcare delivery. The corresponding results are shown in Fig. 4. While diagnostic accuracy demonstrates a relatively small drop in accuracy, the patient agent follow-up perceptions tell a different story. Broadly, we find that most patient cognitive biases did not have a strong effect on any of the patient perceptions when compared to an unbiased patient agent except for in the case of self-diagnosis, which had sizeable drops in confidence (4.7 points) and consultation (2 points), and a minor drop in compliance (1 point). However, implicit biases had a profound effect on all three categories of patient perception, with education bias consistently reducing patient perception across all three categories.

We found that between the implicit biases, sexual orientation bias had the lowest effect on patient perceptions, followed by racial bias and gender bias. For patient confidence, gender bias is followed by religious, socioeconomic, cultural, and education biases. For patient compliance and patient consultation, gender bias is followed by cultural, socioeconomic, religious, and education biases. While it is not quantifiable, we decided to ask two biased patient agents who provided low rating with education and gender biases for compliance *why* they provided low ratings. These patient agents had the same symptoms and diagnosis and only differed in bias presentation.

It is important to note that the patient agents used in our study are simulated by language models, which may not fully capture the complexity and variability of real human patients. As such, the confidence, compliance, and consultation ratings provided by these agents may not perfectly reflect real-world patient perceptions, rather, provide insight into how real-world bias can be studied through clinical simulations. While we acknowledge that metrics derived from simulated agents are not a direct proxy for real human patient experiences, we present this analysis to provide initial insights into how biases might manifest in clinical interactions and affect patient trust and compliance within a simulated environment. The following results should be interpreted as an exploration of simulated agent behavior, not a definitive measure of human perception.

### Specialist and multilingual cases

**Fig. 5:**
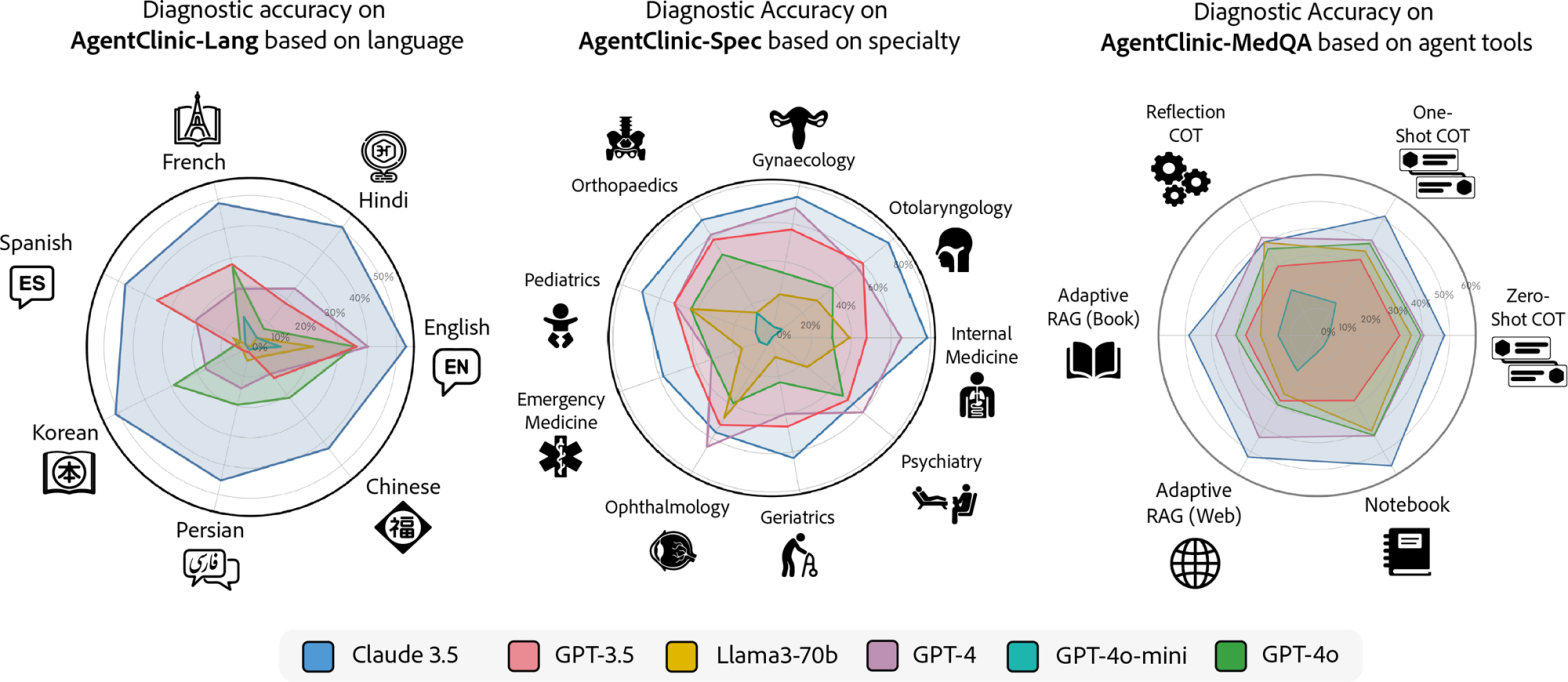
Specialist, multilingual, and tool usage. Diagnostic accuracy based on language (Left), based on medical specialty (Middle), and based on agent tools (Right).

We now focus on specialist rather than general medical cases. Specialist cases use reports that are derived from datasets focusing on specific medical specialties (e.g., internal medicine, psychiatry) and are designed to simulate complex diagnostic scenarios requiring in-depth expertise. In contrast, general QA tasks involve static, single-turn multiple-choice questions such as those found in medical licensing exams. An analysis of language model performance across nine medical specialties reveals significant differences in diagnostic accuracy (Fig. 5). Claude 3.5 achieved the highest overall performance with an average accuracy of 66.7%, excelling in Internal Medicine (78.3%), Otolaryngology (76.7%), and Gynecology (74.3%). GPT-4 demonstrated strong performance in Gynecology (68.5%) and Ophthalmology (65.2%) but showed reduced accuracy in Emergency Medicine (32.3%) and Geriatrics (40%). GPT-3.5 outperformed some newer models in specific areas, such as Emergency Medicine (41.9%), and maintained an average accuracy of 51.8%. In contrast, Llama3-70b and GPT-4o-mini consistently underperformed across most specialties, highlighting a significant gap between language models in handling specialist medical tasks.

The variations in performance across different medical domains suggest that certain specialties present more challenges for language models. Specialties like Internal Medicine and Gynecology generally saw higher accuracy rates, which contrasts with existing medical QA literature that identifies Psychiatry and Otolaryngology as the least challenging specialties^17^. This discrepancy may indicate inherent difficulties in diagnosing diseases through dialogue-based interactions as opposed to multiple-choice question formats. Additionally, specialist cases sourced from MedMCQA exhibited higher average accuracy compared to non-specialist cases from MedQA, which differs from reported multiple choice evaluations where specialist QAs typically have lower performance^3^.

We also explore the impact of language on diagnostic accuracy using AgentClinic-Lang, which encompassed seven languages: English, Chinese, French, Spanish, Hindi, Persian, and Korean (Fig. 5). The generated cases were manually inspected by native speakers to ensure linguistic accuracy. The speakers were fluent in the target language but were not required to have formal medical training. Six multilingual models were evaluated, including GPT-4, GPT-4o, GPT-4o-mini, GPT-3.5, Llama 3 70B-Instruct, and Claude 3.5 Sonnet. Overall, all models performed best in English, with performance varying significantly across other languages. Claude 3.5 Sonnet stood out by maintaining high and consistent performance across all languages, achieving an average accuracy of 48.4%, which is more than double that of the next best model, GPT-4, at 20.9%.

Other models exhibited considerable variability in performance across different languages. For example, GPT-4’s accuracy ranged from 11.21% in Chinese to 40.18% in English, while GPT-4o’s performance spanned from 3.73% in Korean to 35.5% in English. GPT-3.5 showed a similar pattern, with accuracies ranging from 1.86% in Persian to 36.3% in English, although it performed relatively well in Korean (35.4%). Llama3-70b and GPT-4o-mini also showed low accuracies across most languages, with Llama3-70b’s highest accuracy being 47.8% in Ophthalmology and GPT-4o-mini achieving a maximum of 14.7% in Orthopedics. Notably, Chinese remained a challenging language for most models, except for Claude 3.5 Sonnet, which maintained relatively high accuracy levels across all tested languages.

### Human dialogue ratings

AgentClinic introduces an evaluation for LLMs patient diagnosis. However, the realism of the actual dialogue itself has yet to be evaluated. To clarify the volume of rated data, the three human clinician evaluators (individuals with MDs) rated a total of *N* = 20 complete dialogue simulations, randomly selected from the English-language AgentClinic-MedQA dataset. We present results from three human clinicians (individuals with MDs) who rated dialogues from 20 agents on English-language AgentClinic-MedQA cases from 1 to 10 across four axes:

1. **Doctor**: How realistically the doctor played the given case.
2. **Patient**: How realistically the patient played the given case.
3. **Measurement**: How accurate & realistic the measurement reader reflects actual case results.
4. **Empathy**: How empathetic the doctor agent was in their conversation with the patient agent.

We find the average ratings from evaluators for each category as follows: Doctor 6.2, Patient 6.7, Measurement 6.3, and Empathy 5.8. We find from review comments that the lower rating for the doctor agent stems from several points such as providing a bad opening statement, making basic errors, overly focusing on a particular diagnostic, or not being diligent enough. For the patient agent, comments were made on them being overly verbose and unnecessarily repeating the question back to the doctor agent. The measurement agent was noted to occasionally not return all of the necessary values for a test (e.g., the following comment *"Measurement only returns Hct and Lc for CBC. Measurement did not return Factor VIII or IX levels / assay”*). Regarding empathy, the doctor agent adopts a neutral tone and does not open the dialogue with an inviting question. Instead, it cuts right to the chase, immediately focusing on the patient’s current symptoms and medical history.

### Diagnostic accuracy in a multimodal environment

Many types of diagnoses require the physician to visually inspect the patient, such as with infections and rashes. Additionally, imaging tools such as X-ray, CT, and MRI provide a detailed and rich view into the patient, with hospitalized patients receiving an average of 1.42 diagnostic images per patient stay^22^. However, the previous experiments in this work and prior work^23^ provided measurement results through text, and did not explore the ability of the model to understand visual context. Here, we evaluate four multimodal LLMs, Claude 3.5 Sonnet, GPT-4o, GPT-4 and GPT-4o-mini, in a diagnostic settings that require interacting through both dialogue as well as understanding image readings. We collect our questions from New England Journal of Medicine (NEJM) case challenges. These published cases are presented as diagnostic challenges from real medical scenarios, and have an associated pathology-confirmed diagnosis. We randomly sample 120 challenges from a sample of 932 total cases for AgentClinic-NEJM. While for human viewers, these cases are provided with a set of multiple choice answers, we chose to not provide these options to the doctor agent and instead keep the problems open-ended.

The goal of this experiment is to understand how accuracy differs when the LLM is required to understand an image in addition to interacting through patient dialogue. We allow for 20 doctor inferences, and condition the patient in the same way as previous experiment with the addition of an image that is provided to the doctor agent. The mechanism for receiving image input in AgentClinic-NEJM is supported in two ways: provided initially to the doctor agent upon initialization and as feedback from the instrument agent upon request.

**Fig. 6:**
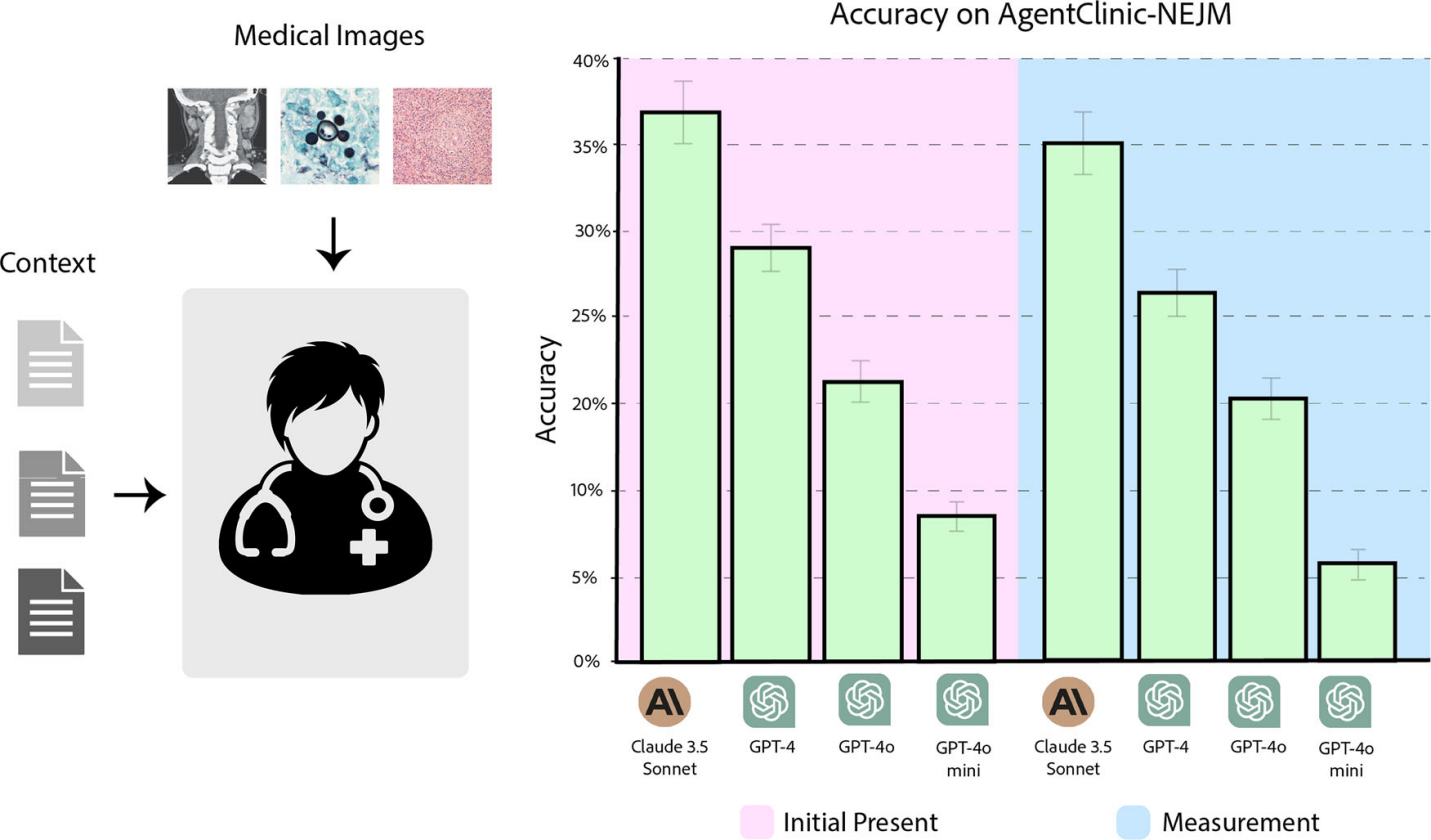
Accuracy of Claude 3.5 Sonnet, GPT-4, GPT-4o, and GPT-4o-mini on AgentClinic-NEJM with multimodal text and language input. (Pink) Accuracy when the images are presented as initial input. (Blue) Accuracy when images must be requested from the image reader.

When the image is provided initially to the doctor agent, across 120 multimodal patient settings we find that Claude 3.5 Sonnet obtains an accuracy of 37.2 ± 2.2, GPT-4 obtains 27.7% ± 2.0, GPT-4o obtains 21.4% ± 1.7 and GPT-4o-mini obtain an accuracy of 8.0% ± 1.2 (Fig. 6). We also find that for the provided *incorrect* responses from GPT-4, the answer that was provided was among those listed in the multiple choice options 60% of the time. In the case of when images are provided upon request from the instrument agent we find that Claude 3.5 Sonnet obtains an accuracy of 35.4 ± 2.4, GPT-4 obtains 25.4% ± 2.1, GPT-4o obtains 19.1% ± 1.4 and GPT-4o-mini obtains 6.1% ± 1.2 (Fig. 6).

### How does limited time affect diagnostic accuracy?

One of the variables that can be changed during the AgentClinic-MedQA evaluation is the amount of interaction steps that the doctor is allotted. For other experiments we’ve demonstrated, the number of interactions between the patient agent and doctor agent was set to *N* = 20. Here, both the doctor and the patient agent can respond 20 times, producing in total 40 lines of dialogue. By varying this number, we can test the ability of the doctor to correctly diagnose the patient agent when presented with limited time (or a surplus of time).

We test decreasing the time to *N* = 10 and *N* = 15 as well as increasing the time to *N* = 25 and *N* = 30. We find that accuracy decreases from 52% when *N* = 20 to 25% when *N* = 10 and 38% when *N* = 15. This large drop in accuracy is partially because of the doctor agent not providing a diagnosis at all, perhaps due to not having enough information. When *N* is set to a larger value, *N* = 25 and *N* = 30, the accuracy actually *decreases* slightly from 52% when *N* = 20 to 48% when *N* = 25 and 43% when *N*=30. This is likely due to the growing input size, which can be difficult for language models.

In real medical settings, one study suggest that the average family physician asks 3.2 questions and spends less than 2 minutes before arriving at a conclusion^24^. It is worth noting that interaction time can be quite limited due to the relative low-supply and high-demand of doctors (in the US). In contrast, deployed language agents are not necessarily limited by time while interacting with patients. So, while limiting the amount of interaction time provides an interesting scenario for evaluating language models, it may also be worth exploring the accuracy of LLMs when N is very large.

### Does the patient language model affect accuracy?

Here, we explore whether the patient agent model plays a role in diagnostic accuracy. The primary quantitative comparisons for this analysis are reported in the earlier model-comparison results and are summarized in Fig. 2 (middle panel). This subsection focuses on qualitative behavioral differences observed in the patient-agent responses.

Inspecting the dialogues, we observed that GPT-3.5 patient agents more frequently repeat or paraphrase the doctor’s question without adding new information. For example, consider the following dialogue snippet: “*Doctor: Have you experienced any muscle twitching or cramps? Patient: No, I haven’t experienced any muscle twitching or cramps*.” In contrast, GPT-4 patient agents more frequently provide novel symptomatic details in response to similar prompts. For example: “*Doctor: Have you had any recent infections, like a cold or the flu, before these symptoms started? Patient: Yes, I’ve had a couple of colds back to back and a stomach bug in the last few months*.” These behavioral differences may contribute to differences in diagnostic performance by altering the amount of clinically relevant information surfaced during dialogue.

When a GPT-3.5 doctor agent interacts with a GPT-4 patient agent, the accuracy comes out to 38%, but when a GPT-3.5 doctor interacts with a GPT-3.5 patient agent the accuracy comes out to a very similar value of 37% which would be expected to be much lower. We suspect that cross-communication between different language models provides an additional challenge. Recent work supports this hypothesis by demonstrating a linear relationship between self-recognition capability and the strength of self-preference bias^18^. This work shows that language models can recognize their own text with high accuracy, and display disproportionate preference to that text, which may suggest there is an advantage for doctor models which have the same LLM acting as the patient agent.

### Coverage of MedQA versus AgentClinic-MedQA

To better understand the performance differences between MedQA and AgentClinic-MedQA, we conducted an analysis to quantify the amount of relevant patient information obtained by the doctor agents in each setting. Specifically, we focused on measuring *coverage*—the proportion of relevant information successfully extracted by the doctor agent through dialogue with the patient agent or through measurement interactions.

For this analysis, we selected a sample of MedQA cases and their corresponding AgentClinic-MedQA simulations, using GPT-4 as the doctor agent. In MedQA, all relevant patient information, such as symptoms, medical history, and test results, is provided upfront in a static format. In contrast, AgentClinic-MedQA requires the doctor agent to dynamically gather this information through interactions. To evaluate coverage, we manually reviewed the dialogues in AgentClinic-MedQA and determined whether the doctor agent extracted each piece of relevant information identified in the MedQA cases. Coverage was calculated as the ratio of extracted information to the total relevant information available in the MedQA cases.

Our findings revealed that the average coverage in AgentClinic-MedQA was 67%. Furthermore, the coverage was notably higher (72%) in cases where the doctor agent provided a correct diagnosis, compared to 63% in cases where the diagnosis was incorrect. These results suggest that the ability to extract more complete information is a key factor in accurate diagnoses in AgentClinic-MedQA. The discrepancy in diagnostic accuracy between MedQA and AgentClinic-MedQA can likely be attributed to the additional complexity of acquiring information in the latter, as opposed to the static format of the former. Based on our findings, we recommend the following for future evaluations using AgentClinic to identify capable doctor agents. First, performance should not be assessed against a single patient agent model. As our results show, a doctor agent’s accuracy can vary when interacting with different patient models (e.g., GPT-4 vs. GPT-3.5). We suggest evaluating against a panel of patient agents with varying capabilities to assess robustness. Second, testing for resilience to bias is critical. We observed that some models, like GPT-4, maintain high accuracy under bias, while others, like Mixtral-8 × 7B, see significant degradation. Therefore, we recommend running evaluations across a suite of cognitive and implicit biases to identify models that are not only accurate but also robust. Finally, tool-use capability is a key differentiator; evaluating how effectively a model can leverage tools like adaptive RAG or a notebook is essential for determining its potential in a real-world clinical workflow.

## Discussion

In this work, we present AgentClinic: a multimodal agent benchmark for simulating clinical environments. We design 120 multimodal language agents which require an understanding of both language and images and 215 language agents based on cases from the USMLE. We also introduce 260 patient cases from 9 medical specialties and 749 patient cases from 7 multilingual environments. We instructed these agents to exhibit 23 different biases, with either the doctor or patient presenting bias. Notably, models like GPT-4 demonstrated resilience to cognitive and implicit biases, maintaining high diagnostic accuracy, while others like Mixtral-8 × 7B experienced significant performance degradation. We also find that doctor and patient biases can reduce diagnostic accuracy, and that the patient has a lower willingness to follow up with treatment, reduced confidence in their doctor, and lower willingness to have a follow-up consultation in the presence of bias. Tool use, such as adaptive retrieval and reflection cycles, revealed substantial differences in LLMs’ abilities to enhance their performance, with models like Llama 3 showing up to 19.7% improvement.

Our work presents only a simplified clinical environment that includes agents representing a patient, doctor, measurements, and a moderator. One potential limitation of the presented workflow comes from the use of an LLM for determining accuracy via the moderator agent (albeit, provided a ground truth). Recent research^25^ has shown that strong LLM judges like GPT-4 can match both controlled and crowd-sourced human preferences well, achieving over 80% agreement, which is the same level of agreement between humans, indicating the use of an LLM may not be limiting. Additionally, while the measurement agent adds a flexible interface for gathering medical exam results, its reliance on using an LLM to provide results may introduce errors or hallucinations, which could be mitigated through a database or SQL tool. In future work, we will consider including additional critical actors such as nurses, the relatives of patients, administrators, and insurance contacts. There may be additional advantages to creating agents that are embodied in a simulated world^26,27^, so that physical constraints can be considered, such as making decisions with limited hospital space. Additionally, future work could explore the role of demographic biases, such as race and gender. It is also important to note that the human data used for baselining and rating were based on a small sample size (*N* = 3 human clinicians). Consequently, these findings should not be interpreted as generalizable to the broader population of clinicians.

Another limitation of our evaluations is the uncertainty regarding the training data of proprietary models like GPT-4 and Claude 3.5. It’s possible that these models were trained on datasets like MedQA, potentially giving them an unfair advantage due to data leakage. However, by focusing on novel interaction patterns, such as query frequency and tool-use sequences within the dynamic AgentClinic environment, our findings are inherently more robust to the data leakage concerns that affect studies using static, well-established clinical vignettes. If significant verbatim overlap existed, a retrieval-augmented generation (RAG) system could, in principle, achieve strong performance by retrieving memorized content rather than demonstrating genuine reasoning. While our multi-turn, multimodal scenario transformation is designed to reduce this risk by breaking direct question-answer mappings, we cannot entirely exclude it. Future work will include a baseline evaluation with a standard RAG+LLM pipeline to better quantify the degree to which AgentClinic requires reasoning beyond retrieval. While our results showing that MedQA performance is not predictive of AgentClinic-MedQA accuracy (Fig. 3) provides evidence that this may not be an issue, it is possible that GPT-4/4o/3.5 or Claude 3.5 could have been trained on the MedQA test set. Currently, Mixtral-8 × 7B^28^ and Llama 2-70B-Chat^29^ do not report training on the MedQA test or train set. Future work should focus on developing evaluation datasets that are less likely to have been included in pre-training corpora or on collaborating with model developers to ensure fair assessments. Another limitation for the experiments on varying the patient LLM suggest that there may be an advantage for LLMs which act as both the patient and the doctor agent, because LLMs are able to recognize their own text with high accuracy, and display disproportionate preference to that text^18^. While our moderator agent is based on an LLM judge shown to have high agreement with humans, the sensitivity of our benchmark results to variations in the moderator agent’s own model or system prompt was not explored. This represents a potential source of variability and an avenue for future investigation. Additionally, for the human dialogue ratings, the three evaluators were all certified with MDs (ER, JJ, MM), but their specific medical specialties were not controlled for, representing a potential area for more targeted evaluation in future studies.

Future work could expand on our initial coverage analysis to develop a standardized information elicitation score for different patient agents and bias configurations. This would allow for a deeper understanding of the relationship between an agent’s ability to gather information and its diagnostic performance. Another consideration in our study is the use of LLMs to simulate all interacting agents. While this approach enables controlled and scalable experimentation, it introduces the possibility that model-specific behaviors or inherent architectural priors could influence the outcomes. For instance, the reasoning patterns or safety training of the LLM used for the moderator might interact in unforeseen ways with the LLM used for the clinician agent. Although we validated each component’s performance on its core task, the emergent dynamics of the multi-agent system remain an area for further research. Future studies should explore the impact of using diverse model architectures for different roles within the simulation. Finally, key directions for future work include not only expanding the range of biases and clinical scenarios but also performing a deeper analysis of the emergent “cross-model communication” effects observed here, which could yield significant insights into the collaborative and interpretive capabilities of AI agents.

We recognize that the tool-use commands in AgentClinic are abstractions of real-world clinical workflows. For example, requesting a laboratory test or imaging study is simplified to a single structured command, whereas in actual practice such actions involve multiple steps, stakeholders, and contextual constraints. This level of abstraction was chosen deliberately to standardize agent interactions and make evaluation tractable across specialties and languages. Nevertheless, we acknowledge that this simplification limits ecological validity. As future work, we plan to extend AgentClinic with a more fine-grained and hierarchical set of tools that better approximate the complexity of real diagnostic and information-gathering procedures, including workflow dependencies and resource constraints.

Previous benchmarks like AMIE^23^, SAPS^30^, and CRAFT-MD^31^ focus on dialogue-based evaluations but lack multimodal capabilities and do not simulate real-world biases, tool usage, multilingual, or specialist cases. MedAgents^32^ emphasizes QA improvement through agent collaboration but does not simulate patient interactions or decision-making processes. AgentClinic advances the field by providing an interactive, multimodal environment with bias simulation and tool integration, offering a more comprehensive evaluation platform for medical AI systems.

Overall, we believe that LLMs need to be examined with novel evaluation strategies that go well beyond static question-answering benchmarks. With this work, we take a step towards building more interactive, operationalized, and dialogue-driven benchmarks that scrutinize the sequential decision making ability of language agents in various challenging and multimodal clinical settings.

## Methods

### Agent details

The patient agent has knowledge of a provided set of symptoms and medical history, but lacks knowledge of the what the actual diagnosis is. The role of this agent is to interact with the doctor agent by providing symptom information and responding to inquiries in a way that mimics real patient experiences.

The function of the measurement agent is to provide realistic medical readings for a patient given their particular condition. This agent allows the doctor agent to request particular tests to be performed on the patient. The measurement agent is conditioned with a wide range of test results from the scenario template that are expected of a patient with their particular condition. For example, a patient with Acute Myocardial Infarction might return the following test results upon request “*Electrocardiogram: ST-segment elevation in leads II, III, and aVF., Cardiac Markers: Troponin I: Elevated, Creatine Kinase MB: Elevated, Chest X-Ray: No pulmonary congestion, normal heart size*”. A patient with, for example, Hodgkin’s lymphoma, might have a large panel of laboratory parameters that present abnormal (hemoglobin, platelets, white blood cells (WBC), etc).

The doctor agent serves as the primary object that is being evaluated. This agent is initially provided with minimal context about what is known about the patient as well as a brief objective (e.g., “*Evaluate the patient presenting with chest pain, palpitations, and shortness of breath*”). They are then instructed to investigate the patients symptoms via dialogue and data collection to arrive at a diagnosis. The doctor agent is also able to request test results from the measurement agent, specifying which test is to be performed (e.g., Chest X-Ray, EKG, blood pressure). When test results are requested, this also is counted toward the number of questions remaining. In order to simulate realistic constraints, the doctor agent is provided with a limited number of questions that they are able to ask the patient^24^. Here, the doctor agent is provided with a limited number of interaction turns, which was set to *N* = 20 for most experiments. A single interaction turn consists of one response from the doctor agent and one response from either a tool or from the measurement or patient agent depending on the doctor agent response. After the final turn, the agent must provide its diagnosis, which is then evaluated by the moderator.

The function of the moderator is to determine whether the doctor agent has correctly diagnosed the patient at the end of the session using a ground truth accuracy label provided to the moderator. This agent is necessary because the diagnosis text produced by the doctor agent can be quite unstructured depending on the model, and must be parsed appropriately to determine whether the doctor agent arrived at the correct conclusion. For example, for a correct diagnosis of “Type 2 Diabetes Mellitus,” the doctor might respond with the unstructured dialogue: “*Given all the information we’ve gathered, including your symptoms, elevated blood sugar levels, presence of glucose and ketones in your urine, and unintentional weight loss I believe a diagnosis of Type 2 Diabetes with possible insulin resistance is appropriate*,” and the moderator must determine if this diagnosis was correct. Specifically, the moderator agent’s instructions are: “Here is the correct diagnosis: [correct diagnosis]. Here was the doctor dialogue: [doctor agent's diagnosis]. Are these the same? You are responsible for determine if the correct diagnosis and the doctor diagnosis are the same disease. Please respond only with Yes or No. Nothing else”. This evaluation may also become more complicated, such as in the following example diagnosis: “*Given your CT and blood results, I believe a diagnosis of PE is the most reasonable conclusion*,” where PE (Pulmonary Embolism) represents the correct diagnosis abbreviated.

### Patient perception metrics

After each patient–doctor interaction, the patient agent produced three scalar ratings on a 1–10 Likert-type scale: (i) confidence in the doctor’s assessment, (ii) compliance (self-reported likelihood of following the recommended therapy), and (iii) consultation willingness (likelihood of consulting the same doctor again). No semantic descriptors were provided for individual integer levels in the system prompt (e.g., no explicit definition distinguishing a 6 from a 7). The ratings were therefore generated based on the evaluating model’s internal calibration to the dialogue context. No additional inter-run reliability analysis was performed for these scalar ratings. Analyses emphasize relative differences between experimental conditions (e.g., biased vs. unbiased) rather than interpretation of absolute rating values.

### Diagnostic accuracy in a multimodal environment

To more rigorously test the models’ diagnostic reasoning in an unprompted setting, we chose to not provide these options to the doctor agent and instead keep the problems open-ended. The agent’s open-ended diagnosis was then evaluated using the standard moderator agent, which compared the output to the pathology-confirmed ground truth diagnosis provided in the original NEJM case, as established by clinicians with domain expertise. The faithfulness of LLMs in establishing judgment is established in previous works^33–35^.

### Bias details

Cognitive biases are systematic patterns of deviation from norm or rationality in judgment, where individuals draw inferences about situations in an illogical fashion^36^. These biases can impact the perception of an individual in various contexts, including medical diagnosis, by influencing how information is interpreted and leading to potential errors or misjudgments. The effect that cognitive biases can have on medical practitioners is well characterized in literature on misdiagnosis^37^. In this work, we introduce cognitive bias prompts in the LLM system prompt for both the patient and doctor agents. For example, the patient agent can be biased toward believing their symptoms are pointing toward them having a particular disease (e.g., *cancer*) based on their personal internet research. The doctor can also be biased toward believing the patient symptoms are showing them having a particular disease based on a recently diagnosed patient with similar symptoms (recency bias).

Implicit biases are associations held by individuals that operate unconsciously and can influence judgments and behaviors towards various social groups^38^. These biases may contribute to disparities in treatment based on characteristics such as race, ethnicity, gender identity, sexual orientation, age, disability, health status, and others, rather than objective evidence or individual merit. These biases can affect interpersonal interactions, leading to disparities in outcomes for the patient, and are well characterized in the medical literature^38–40^. Unlike cognitive biases, which often stem from inherent flaws in human reasoning and information processing, implicit biases are primarily shaped by societal norms, cultural influences, and personal experiences. In the context of medical diagnosis, implicit biases can influence a doctor’s perception, diagnostic investigation, and treatment plans for a patient. Implicit biases of patients can affect their trust—which is needed to open up during history taking—and their compliance with a doctor’s recommendations^39^. Thus, we define implicit biases for both the doctor and patient agents.

### Model details

Briefly, we discuss the details of each model below starting with language models followed by common language models.

GPT-4 (*gpt-4-0613*) is a large-scale, multimodal LLM which is able to process both image and text inputs. GPT-3.5 (*gpt-3.5-turbo-0613*) is a subclass of GPT-3 (a 170B parameter model)^41^ fine-tuned on additional tokens and with human feedback^42^. Currently, the details regarding the architecture, dataset, and training methodologies of GPT-3.5, GPT-4o (*gpt-4o-2024-05-13*), and GPT-4 have not been not publicly disclosed. However, existing technical documentation indicates that both models are high-performing in medical and biological subjects, with GPT-4 showing superior performance compared to GPT-3.5 in knowledge assessments^3,43^.

Mixtral 8 × 7B is a language model that employs a Sparse Mixture of Experts (SMoE) architecture^28^. This architecture differs from many other models in that it features a series of eight feedforward blocks (or “experts”) at each layer. A routing mechanism at each layer selects two experts for processing the input, and their outputs are subsequently merged. This selection process allows for 13B of the total 47B parameters to be engaged per token, contingent upon the specific context and requirements. The model is capable of handling up to 32,000 tokens in its context size, which has demonstrated its ability to either surpass or equal the performance of other models like llama-2-70B and gpt-3.5 across a range of benchmarks.

Llama is an open-access model developed by Meta, which was trained on 2 trillion tokens from publicly available data^29^. The model comes in various sizes, with parameters ranging from 7 billion to 70 billion. The selection of the 70 billion chat model was based on its superior performance across a range of metrics. Significant efforts were made to align the training process with established safety metrics, leading to improvements in how the model handles adversarial prompting in specified “risk categories.” Notably, this includes the model’s response to requests for advice that it may not be qualified to provide, such as medical advice, which is relevant to the context of this work.

PMC-LLaMA^11^ is an open-source biomedical language model developed to improve performance in medical applications. It employs a two-stage training approach: initially, the model undergoes data-centric knowledge injection using a corpus of 4.8 million biomedical academic papers and 30,000 medical textbooks. Subsequently, it is fine-tuned with a comprehensive instruction dataset comprising 202 million tokens, including medical question-answering, reasoning rationales, and conversational dialogues. The 13B parameter PMC-LLaMA model demonstrates superior performance on various public medical QA benchmarks, even surpassing ChatGPT in certain evaluations.

OpenBioLLM-70B^44^ is a state-of-the-art open-source biomedical language model developed by Saama AI Labs. Built upon Meta’s Llama 3 architecture, it is fine-tuned using a diverse corpus of high-quality biomedical data and employs advanced techniques like Direct Preference Optimization (DPO). The model exhibits superior performance across multiple biomedical benchmarks, outperforming other open-source models and even larger proprietary models like GPT-4 and Med-PaLM-2.

MedLLaMA3^45^ is a fine-tuned large language model based on Meta’s LLaMA 3 architecture, specifically tailored for medical applications. On May 2024, MedLLaMA3 ranked second on the HuggingFace MedLLM Leaderboard, showcasing its exceptional performance in medical-related natural language processing tasks

Meditron-70B^12^ is a 70-billion-parameter medical language model developed by the EPFL LLM Team. It is adapted from LLaMA 2-70B through continued pretraining on a comprehensively curated medical corpus, including selected PubMed articles, abstracts, and internationally recognized medical guidelines.

### Tool descriptions

Chain-of-thought reasoning is a technique that allows language agents to articulate their reasoning process step-by-step when solving complex problems^46,47^. By breaking down the problem-solving process into smaller, logical steps, agents can better handle intricate tasks, improve their reasoning capabilities, and provide more transparent and interpretable solutions. Zero-shot CoT^47^ prompts the model to use this reasoning without examples, while one-shot CoT^46^ provides a single example to guide the model’s thought process, potentially leading to improved performance in complex reasoning tasks. Our implementation of one-shot CoT employed a few-shot prompting strategy with k=1 examples. This example was selected from a repository of successfully solved cases based on semantic similarity between the current problem and the previously solved cases in the agent’s memory.

Experiential learning in the context of AI agents refers to the ability to accumulate knowledge and insights from past interactions and apply them to future tasks^48^. This technique allows agents to improve their performance over time by learning from successes, failures, and feedback received during previous engagements. This was previously explored in Agent Hospital^27^ through an experience retrieval system. By maintaining a form of “memory” or knowledge base that updates through interaction, agents can become better at handling similar situations, adapting to user preferences, and providing increasingly relevant and accurate responses as they gain more “experience” in their operational domain. In our work, we enable the doctor agent to use a memory “notebook” which persists across patients. Here, the doctor agent can write useful tips such as the following example from the doctor agent: “*[Note #17] Remember that timing and onset of symptoms can provide valuable diagnostic insights*”.

The experiential learning module enables the agent to learn from its successes. Its operation is as follows: (1) *Verification*: After the agent completes a case, its final answer is compared against the known ground truth. (2) *Reflection*: If the answer is correct, a meta-prompting routine is initiated. This prompt instructs the model to reflect on its own successful Chain-of-Thought and synthesize a concise “takeaway”—a summary of the effective reasoning strategy. (3) *Memory Storage*: This takeaway, along with the original case query, is stored in a vector database that serves as the agent’s experiential memory. (4) *Experiential Retrieval*: For new cases, the agent first queries this memory to retrieve the most semantically similar takeaways from its past successes. These are then inserted into its prompt as dynamic, highly relevant few-shot examples. This entire process is a form of in-context learning; it does not involve updating the model’s weights and is distinct from standard RAG, as retrieval occurs from a dynamic memory of solved cases, not a static external knowledge base.

To enable the doctor agent to research medical information, we introduce a method using (an adaptive form of retrieval augmented generation (RAG) from medical sources. RAG involves retrieving relevant information from a knowledge base and using it to augment the input of an LLM during the generation process^49^, thereby improving the factual consistency of generated text by grounding it in retrieved information. Conventional RAG methods passively retrieve information at every inference call without allowing the agent to control the timing or content of retrieval. To address this limitation, we employ adaptive retrieval^50,51^, which enables the LLM to actively determine when and what information to retrieve. Our implementation provides the doctor agent with two categories of retrieval: internet and textbook databases. The internet database contains material from sources such as PubMed(https://pubmed.ncbi.nlm.nih.gov/) research articles, StatPearls(https://www.statpearls.com/)—a database of articles written for healthcare professionals—and Wikipedia articles on various medical topics. The textbook database includes 18 medical textbooks commonly used by medical students in the United States^52^. The doctor agent can retrieve information by issuing commands similar to requesting medical scans, using the format: *"Research [database] [search query]”*. For example, the command *"Research textbooks ’What are the symptoms of myasthenia gravis?”’* prompts the retrieval of relevant information.

## Data Availability

The data from the AgentClinic benchmark is open under an MIT license. All data is available at github.com/SamuelSchmidgall/AgentClinic. MIMIC-IV based datasets can be requested by the authors upon completion of PhysioNet training, as per the PhysioNet guidelines.

## Code availability

The AgentClinic benchmark is open-source. All code, prompts, and evaluation scripts are available under an MIT license at github.com/SamuelSchmidgall/AgentClinic.
